# Association of *MUC1 5*640G>A and *PSCA* 5057C>T polymorphisms with the risk of gastric cancer in Northern Iran

**DOI:** 10.1186/s12881-020-01085-z

**Published:** 2020-07-13

**Authors:** Reza Alikhani, Ali Taravati, Mohammad Bagher Hashemi-Soteh

**Affiliations:** 1 Department of Molecular and Cell Biology, Faculty of Basic Sciences, University of Mazandaran, Babolsar, Mazandaran Iran; 2 Immunogenetic Research center, Molecular and Cell Biology Research Center, Medical Faculty, Mazandaran University of Medical Sciences, Sari, Mazandaran 48166-13485 Iran

## Abstract

**Background:**

Gastric cancer is one of the four most common cancer that causing death worldwide. Genome-Wide Association Studies (GWAS) have shown that genetic diversities *MUC1* (Mucin 1) and *PSCA* (Prostate Stem Cell Antigen) genes are involved in gastric cancer. The aim of this study was avaluating the association of rs4072037G > A polymorphism in *MUC1* and rs2294008 C > T in *PSCA* gene with risk of gastric cancer in northern Iran.

**Methods:**

DNA was extracted from 99 formalin fixed paraffin-embedded (FFPE) tissue samples of gastric cancer and 96 peripheral blood samples from healthy individuals (sex matched) as controls. Two desired polymorphisms, 5640G > A and 5057C > T for *MUC1* and *PSCA* genes were genotyped using PCR-RFLP method.

**Results:**

The G allele at rs4072037 of *MUC1* gene was associated with a significant decreased gastric cancer risk (OR = 0.507, 95% CI: 0.322–0.799, *p* = 0.003). A significant decreased risk of gastric cancer was observed in people with either AG vs. AA, AG + AA vs. GG and AA+GG vs. AG genotypes of *MUC1* polymorphism (OR = 4.296, 95% CI: 1.190–15.517, *p* = 0.026), (OR = 3.726, 95% CI: 2.033–6.830, *p* = 0.0001) and (OR = 0.223, 95% CI: 0.120–0.413, *p* = 0.0001) respectively. Finally, there was no significant association between the PSCA 5057C > T polymorphism and risk of gastric cancer in all genetic models.

**Conclusion:**

Results indicated that the *MUC1* 5640G > A polymorphism may have protective effect for gastric cancer in the Northern Iran population and could be considered as a potential molecular marker in gastric cancer.

## Background

Gastric cancer is the fourth most common cancer worldwide and the second leading cause of cancer-related death [1]. The prevalence of gastric cancer varies among different geographic populations around the world [2]. More than 70% of deaths from stomach cancer occur in developing countries [3, 4]. Northern and northwestern regions of Iran are at high risk for gastric cancer [2]. In addition, the mortality rate from stomach cancer is the first cause of death due to cancer in both sexes in Iran [5, 6]. From 2000 to 2005, the incidence rate was highest in northern Provinces, including Mazandaran, Golestan, and Ardabil [2]. It is believed that different environmental risk factors like alcohol consumption, smoking habits, diet and infectious agents (Helicobacter pylori) are involved in the development of gastric cancer [7–9], but a genetic predisposition to stomach cancer is unknown yet. Previous studies suggested an association between *MUC1* and stomach [10], colon [11], lung [12], ovarian [13], breast [14], pancreas [15], and thyroid cancers [16]. In addition, Genome-Wide Association Studies (GWAS) proposed genetic diversities like rs4072037G > A (5640G > A) in *MUC1* gene and rs2294008C > T (5057C > T) polymorphisms in *PSCA* (Prostate Stem Cell Antigen) gene as genetic risk factors in gastric cancer [17].

*MUC1* (Mucin 1) gene is located on chromosome 1q21 and includes seven exons [18]. This gene encodes a membrane-bound glycoprotein with the transmembrane domain, which is attached to the upper surface of the gastrointestinal epithelium. It plays important roles in protecting epithelial surfaces against external agents [19, 20]. MUC1 protein in tumor cells or tumor-associated MUC1 (TA-MUC1) differs from MUC1 expressed in normal cells, with regard to its cellular distribution, biochemical features, and function. TA-MUC1 is hypoglycosylated and overexpressed in a variety of cancers, which plays a crucial role in progression of the disease [21]. It is believed that hypoglycosylation may potentiate *MUC1* oncogenic signaling by decreasing its cell surface levels and increasing intracellular accumulation [21, 22].

*PSCA* gene is located on chromosome 8q24.3, which encodes a glycosylphosphatidyl inositol (GPI)-anchored protein with 114 amino acids. *PSCA* gene product has important role in proliferation, cell adhesion, and survival [23, 24]. PSCA is expressed in the epithelial cells of the bladder, stomach, kidney, skin, and esophagus. The function of PSCA in normal cells and carcinogenesis is not clearly known [25]. Several reports indicated that *PSCA* is associated with various cancers, such as prostate [26], bladder [27], and pancreatic cancer [28]. *PSCA* is up-regulated in the prostate, and pancreas cancers, while in the esophagus and stomach cancers it is down-regulated [29]. 5057C > T is a missense single nucleotide polymorphism (SNP) in 5′-UTR region of *PSCA*. This polymorphism leads to changes in transcriptional activity in the upstream regions of *PSCA* gene [30]. The C allele changes the initiation codon from ATG to the alternative start codon ACG (threonine instead of methionine) and leads to a truncated protein lacking nine amino acids from the N-terminus of PSCA protein. This alteration changes the protein folding and impairs intracellular processes [31].

The aim of this study is also evaluating the association between *MUC1* gene polymorphism, rs4072037G > A and *PSCA* gene polymorphism rs2294008C > T with gastric cancer in northern Iran.

## Methods

### The study population

Ninety nine formalin fixed paraffin-embedded (FFPE) tissue samples (64 males and 35 females, average *age* 67.5 ± 10.9) were collected from gastric cancer patients who referred to the pathology department of the Sari Imam Khomeini hospital from 2009 to 2016 (Table 1) (Mazandaran Province). Tumor characteristics of the patients are summarized in Table 2. In addition, 96 blood samples were collected as control from healthy individuals (57 males and 39 females, average *age* 34.3 ± 7.0) who referred to Novin genetics laboratory of Sari for other reasons except gastric diseases (Table 1). The normal control individuals were not age-matched with patients, but they were selected randomly to find the allele/genotype frequency of the polymorphisms in the local population. Blood samples were collected in EDTA containing tubes and preserved at − 20 °C. This research was approved by the ethics committee in the Mazandaran University of Medical Sciences.

**Table 1:** Demographic characteristics of the cases and controls

Variables	Cases (*n* = 99)		Controls (*n* = 96)		*P*
Age (years) (mean ± SD)	67.5 ± 10.9		34.3 ± 7		0.000
Sex	n	%	n	%	
Male	64	64.65	57	59.38	0.448
Female	35	35.35	39	40.62

**Table 2:** Tumor characteristics, including tumor site, tumor grade, lymphatic invasion, perineural invasion, tumor stage and tumor type

Characteristic		Frequency (%)
**Tumor site**	Cardia	18 (33.3)
	Fundus	0 (0.0)
	Body	11 (20.4)
	Antrum	18 (33.3)
	overlapping	7 (13.0)
	Total	54 (100)
**Grade**	I	13 (18.84)
	II	39 (56.52)
	III	17 (24.64)
	Total	69 (100)
**Lymphatic invasion**	Present	27 (67.5)
	Absent	13 (32.5)
	Total	40 (100)
**Perineural invasion**	Present	28 (54.9)
	Absent	23 (45.1)
	Total	51 (100)
**T**	1	3 (6.1)
	2	19 (38.8)
	3	26 (53.1)
	4	1 (2.0)
	Total	49 (100)
**N**	0	19 (39.58)
	1	18 (37.5)
	2	9 (18.75)
	3	2 (4.17)
	Total	48 (100)
**M**	0	6 (66.7)
	1	3 (33.3)
	Total	9 (100)
**Stage**	I	8 (19.04)
	II	16 (38.09)
	III	15 (35.72)
	IV	3 (7.15)
	Total	42 (100)
**Tumor type**	Diffuse	14 (73.7)
	Intestinal	5 (26.3)
	Total	19 (100)

### DNA extraction

To extracting DNA from paraffin-embedded tissues, a "YTA Genomic DNA Extraction mini kit (Yekta Tajhiz Azma, Iran) was used. Also a standard salting out methods was applied to extract DNA from blood samples, respectively. Finally, the extracted DNA was maintained at − 20 °C until it’s used for further study.

### Genotyping using PCR-RFLP method

**Fig. 1:**
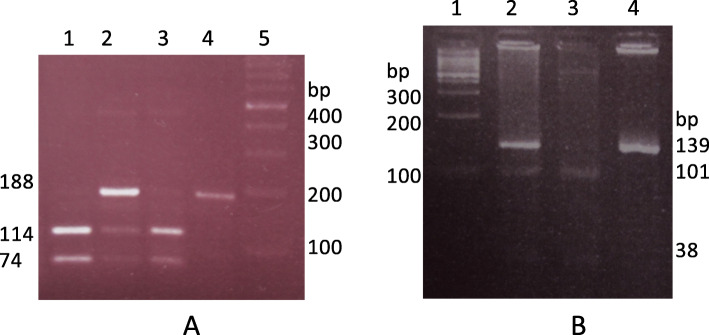
3% agarose gel electrophoresis of the restriction enzyme digestion (**a**) 188 bp PCR fragment of the *MUC1* gene restriction enzyme digestion results using *AIWNI* enzyme; lane 1 and 3 shows homozygous AA, lane 2 heterozygous GA genotype and lane 4 shows homozygous GG genotype respectively, lane 5 shows 100 bp DNA marker (**b**) Restriction enzyme map of the 139 bp PCR fragment of the *PSCA* gene using *NIaIII* enzyme. Lane 1 = 100 bp DNA marker, lane 2 heterozygous CT, lane 3 (101 and 39 bp) shows homozygous TT and lane 4, uncut PCR product shows homozygous CC genotype respectively

PCR (*Polymerase chain reaction*) was applied to amplify exon 2 of *MUC1* and 5′-UTR region of *PSCA* genes, including desired polymorphisms using specific primers (Table 3). Each reaction consists of 2 μl template DNA, 11 μl ready 2x PCR master mix RED (Ampliqon, Denmark), 11 μl distilled water, 0.5 μl of each primer at 25 μM, in a total volume of 25 μl. The PCR reactions were carried out under the following conditions: 94 °C for 5 min, followed by 35 cycles 94 °C for 60 s; 72 °C for 60 s. The annealing temperature of 60 °C for 60 s was used for *MUC1* gene and 56 °C for 60 s used for *PSCA* gene. PCR products were visualized on 1% agarose gel containing SYBR Safe staining. Then, PCR products were subjected to restriction digestion using 6 μl of the PCR products and five units of restriction enzyme. Briefly, 188 bp PCR fragment of the *MUC1* gene was digested using *AIWNI* enzyme; a homozygous GG allele remained uncut, homozygous AA genotype was digested into 114 and 74 bp fragments, and heterozygous GA produced three fragments, 118, 114 and 74 bp, respectively (Fig. 1, a). Also, 139 bp PCR fragment of the *PSCA* gene was digested using a *NIaIII* restriction enzyme, genotype TT was digested to 2 fragments, 101 and 38 bp, genotype CC remained uncut and heterozygous CT showed three fragments, 139, 101 and 38 bp, respectively (Fig. 1, b).

**Table 3:** Primer sequences, PCR size and PCR-RFLP fragments using *AIWNI* and *NIaIII* restriction enzymes for *MUC1* 5640G > A (rs4072037) and *PSCA* 5057C > T (rs2294008) polymorphisms, respectively

SNP	Primer sequence	PCR Size (bp)	Restriction enzyme	Digestion products (bp)		
				Wild-type	Mutant	Heterozygous
*MUC1* 5640G > A (rs4072037)	F: TAAAGACCCAACCCTATGACT	188	*AIWNI*	188	114,74	188,114,74
	R: AGAGTACGCTGCTGGTCATACTC					
*PSCA* 5057C > T (rs2294008	F: GAAACCCGCTGGTGTTGACT	139	*NIaIII*	139	101,38	139,101,38
	R: GCCAAGCCTGCCATCAACAG

### Statistical analysis

The statistical analysis was performed using the SPSS Statistics software (SPSS, Chicago, IL, USA). Different statistical analyses were applied to evaluate the data achieved in this study including Chi-Squre for distributions of demographic characteristics, a logistic regression model for the odds ratio (OR) and confidence interval (CI), respectively. A *p*-value < 0.05 was considered statistically significant. The Hardy–Weinberg equilibrium also was applied to evaluate the frequency of control genotypes against the patient’s samples.

## Results

Demographic and clinical information of 99 patients with gastric cancer and 96 controls are shown in Tables 1 and 2, respectively. Ninety nine patient samples were genotyped for *PSCA* gene (rs2294008) and 91 patient samples for *MUC1* gene (rs4072037) polymorphism. Genotype and allele frequencies of rs4072037 and rs2294008 and their associations with the risk of gastric cancer are presented in Tables 4 and 5, respectively.

**Table 4:** Comparison of genotype and allele frequencies of *MUC1* gene polymorphisms between gastric cancer patients (*n* = 91) and normal controls (*n* = 96) using chi-square analysis

SNP^**a**^	Genotype/Allele	Controls(***n*** = 96)	Cases(***n*** = 91)	OR^**b**^ (95% CI^**c**^)	***P***-value
***MUC1***^***d***^**5640G > A (rs4072037)**	AA	8 (8.4%)	4 (4.1%)	–	–
	AG	27 (28.1%)	58 (63.9%)	4.296 (1.190–15.517)	0.026
	GG	61 (63.5%)	29 (32%)	0.951 (0.265–3.417)	0.938
	AG + GG	88 (91.6%)	87 (95.9%)	0.506 (0.147–1.741)	0.280
	AG + AA	35 (36.5%)	62 (68%)	3.726 (2.033–6.830)	0.0001
	AA+GG	69 (71.9%)	33 (36.1%)	0.223 (0.120–0.413)	0.0001
	A	43 (22%)	66 (36%)	–	–
	G	149 (78%)	116 (64%)	0.507 (0.322–0.799)	0.003

*a Single nucleotide polymorphisms, b* odds ratio, *c* confidence interval, d. Mucin 1

**Table 5:** Comparison of genotype and allele frequencies of PSCA* gene polymorphisms between gastric cancer patients (*n* = 99) and normal controls (*n* = 96) using chi-square analysis

SNP^**a**^	Genotype/Allele	Controls(***n*** = 96)	Cases(***n*** = 99)	OR^**b**^ (95% CI^**c**^)	***P***-value
***PSCA***^***d***^**5057C > T (rs2294008**	CC	63 (65.6%)	62 (62.6%)	–	–
	CT	24 (25%)	29 (29.3%)	1.228 (0.644–2.339)	0.533
	TT	9 (9.4%)	8 (8.1%)	0.903 (0.372–2.492)	0.844
	CT + TT	34 (34.4%)	37 (37.4%)	0.904 (0.505–1.620)	0.735
	CT + CC	90 (90.6%)	91 (91.9%)	1.137 (0.420–3.080)	0.800
	C	150 (78%)	153 (77%)	–	–
	T	42 (22%)	45 (23%)	1.050 (0.652–1.693)	0.840

*a. Single nucleotide polymorphisms, b.* odds ratio, c. confidence interval,* *Prostate stem cell antigen*

The G allele frequency of rs4072037 is 64% in patients with gastric cancer compared with 78% in controls, respectively (Table 4). *MUC1* rs4072037 polymorphism is associated with significant decreased gastric cancer risk in four genetic models: G vs. A (OR = 0.507, 95% CI: 0.322–0.799, *p* = 0.003); heterozygous AG compared with AA (OR = 4.296, 95% CI: 1.190–15.517, *p* = 0.026); dominant model AG + AA vs. GG (OR = 3.726, 95% CI: 2.033–6.830, *p* = 0.0001); and over-dominant model AA+GG vs. G (OR = 0.223, 95% CI: 0.120–0.413, *p* = 0.0001) (Table 4).

In the current study, no significant association was observed between the *PSCA* 5057C > T polymorphism and risk of gastric cancer in all genetic models (T vs. C (OR = 1.050, 95% CI: 0.652–1.693, *p* = 0.840); homozygous TT vs. CC (OR = 0.903, 95% CI: 0.327–2.492, *p* = 0.533); heterozygous CT vs. CC (OR = 1.228, 95% CI: 0.644–2.339, *p* = 0.533); dominant model CT + CC vs. TT (OR = 1.137, 95% CI: 0.420–3.080, *p* = 0.800); and recessive model CC vs. CT + TT (OR = 0.904, 95% CI: 0.505–1.620, *p* = 0.735)). As a result, There was no statistically significant differences between the genotype and allele frequency of *PSCA* (5057C > T) in cases and controls in this study (Table 5).

Furthermore, the result of interaction effects between *MUC1* (rs4072037) and *PSCA* (rs2294008) genotypes with gender is summarized in Table 6.

**Table 6:** Distribution of genotypes *MUC1* (rs4072037) and *PSCA* (rs2294008) polymorphisms according to Sex

Sex	Group	*MUC1* 5640G > A (rs4072037)			*p*-value	*PSCA* 5057C > T (rs2294008)			*p*-value
		GG	GA	AA		CC	CT	TT	
Male	Case	18 (30.5%)	40 (67.8%)	1 (1.7%)	0.0001	41 (64.06%)	17 (26.57%)	6 (9.37%)	0.92
	Control	35 (61.4%)	17 (29.83%)	5 (8.77%)		35 (61.4%)	17 (29.8%)	5 (8.8%)	
Female	Case	11 (34.38%)	18 (56.25%)	3 (9.37%)	0.06	21 (60.0%)	12 (34.3%)	2 (5.7%)	0.25
	Control	24 (61.5%)	12 (30.8%)	3 (7.7%)		28 (71.8%)	7 (17.9%)	4 (10.3%)	

## Discussion

Different previous study demonstrated a decrease incidence in gastric cancer in the world, but it is still one the most frequent cancer in the northern part of Iran [2]. Mazandaran, Golestan, and Ardabil Provinces are high-risk for gastric cancer [2, 32]. Gastric cancer is a multi-factorial disease; a high dietary intake of salt, the high prevalence of Helicobacter pylori (*H. pylori*) infection, smoking and gastroesophageal reflux disease are the environmental factors in the pathogenesis of this disease in Iran [2, 9]. Among genetic risk factors, *MUC1* (5640G > A) and *PSCA* (5057C > T) polymorphisms were reported in several studies earlier [33–36].

MUC1 is a large glycoprotein, which is produced at the apical surface as a transmembrane mucin in most simple epithelia including stomach. MUC1 protein functions are associated with mucins such as hydration of cell surfaces, degradative enzymes, lubrication and protection from microorganisms [37]. MUC1 protein contains three different domains: a large extracellular, transmembrane and a short cytoplasmic domain. The *MUC1* gene in human is comprised of 7 exons that can be alternatively spliced to form different transcripts [37]. At least twelve splice variants of *MUC1* have been described [38]. The SNP rs4072037G > A (5640 G > A) located in the 5′ side of the exon 2 and it is a splicing acceptor site, which produces two different MUC1 transcripts. The “G” allele determines MUC1/B or the variant type 2 and contains the first 27 bp of the exon 2. The “A”allele produce MUC1/A or variant type 3 that have 9 amino acid shorter in the N-terminal domain compared with “G”allele [39–41].

This additional sequence is potentially changing its intracellular trafficking or subsequent processing. The impact of the nine additional amino acids of MUC1/A on the signaling functions and intracellular localization was investigated by Imbert-Fernandez and colleagues [41]. They showed that COS-7 cell line transfected by plasmid containing MUC1/A or MUC1/B had similar protein expression and plasma membrane localization. This study also showed MUC1/B and MUC1/A differs in their ability to modulate tumor necrosis α (TNFα)-induced transcription of IL-1β and IL-8, inducing the basal TGFβ expression; finally they show different inflammatory activities [41].

MUC1 protein could work as a physical barrier and protect the gastric epithelial cells from *H. pylori* by inhibiting its binding to the epithelial cells. The carriers of the short-short (SS) homozygous variant genotype, were at a high risk of *H. pylori* infection, compared to the carriers of the long-long (LL) and long-short (LS) genotypes of *MUC1* variable number of tandem repeats (*VNTR*) [42]. One of the previous study (Miao Li et al., 2013), tested the relation between three polymorphisms (rs4072037 at 1q22, rs13361707 at 5p13, and rs2274223 at 10q23) involved in the increase risk of gastric cancer due to the inflammatory response and *H. pylori* infection [43]. They reported that people infected by *H. pylori,* carrying the genotypes AA rs4072037, CT/CC rs13361707 and AG/GG rs2274223 showed an increased risk of gastric cancer [43].

*Different studies (*Hanze Zhang et al., 2011, J Kupcinskas et al., 2014) have *shown* that the G allele locus on rs4072037 was *significantly associated* with a *decreased gastric cancer risk* [35, 44]. Xinyang Liu et al., 2014 suggested the G allele at rs4072037 of the *MUC1* gene might have a protective rule in people from Asian countries against gastric cancer. Also, they reported that the association was more prevalent in Asian population than in Caucasians [45].

In this study, allelic comparison (G vs. A), genotype comparison (GA vs. AA; GG vs. AA), were done and dominant model (AG + AA vs. GG), recessive model (AA vs. AG + GG), over-dominant model (AA+GG vs. AG) was tested [46]. We found that in this study, the rs4072037 AG genotype was significantly associated with the reduced risk of gastric cancer or it plays a protecting role [odds ratios (OR) = 4.296; 95% confidence interval (CI) = 1.190–15.517, *p* = 0.026 for AG vs. AA].

A study by Hye-Rim Song et al., 2014, with 3245 GC cases and 700 controls suggests that the rs4072037 AG genotype was significantly associated with a reduced risk of gastric cancer [odds ratios (OR) = 0.78; 95% confidence interval (CI) = 0.67–0.91 for AG vs. AA]. They showed an association between the rs4072037 G allele and a reduced risk of gastric cancer [47]. In the other meta-analysis study, with 12,551 cases and 13,436 controls in total from seventeen different case–control studies, suggests different genetic combination (G vs. A; AG vs. AA; GG vs. AA; AG + GG vs. AA) for the *MUC1* rs4072037 polymorphism might decrease the risk of gastric cancer [48]. Also in this study, comparison of the people by ethnicity and the risk of gastric cancer along with the frequency of G allele showed a decrease among Asian population [48].

Another similar study (including 4220 cases and 6384 controls) by Duan F et al., 2014 also evaluated the association between the *MUC1* rs4072037 polymorphism and susceptibility to the cancer [49]. Their study demonstrated that the *MUC1* (rs4072037) polymorphism is associated with risk of cancer in all genetic models (G vs. A; GA vs. AA; GG vs. AA; AG + AA vs. GG; GG vs. AG + AA). They suggested *MUC1* (rs4072037) polymorphism slightly decreased risk of gastric cancer among Asian population, and this was associated with decreased risk with different genotypes except for homozygous recessive (AA) in Caucasian population [49].

In this study, *MUC1* rs4072037 polymorphism was significantly associated with a decreased risk of cancer in all genetic models except for homozygous GG vs. AA and recessive model AG + GG vs. AA (G vs. A: OR = 0.507, 95% CI: 0.322–0.799, *p* = 0.003; AG vs. AA: OR = 4.296, 95% CI: 1.190–15.517, *p* = 0.026; AG + AA vs. GG: OR = 3.726, 95%CI: 2.033–6.830, *p* = 0.0001; AA+GG vs. AG: (OR = 0.223, 95% CI: 0.120–0.413, *p* = 0.0001)). Data from this case-control study indicated that four genetic models G vs. A, heterozygous GA (GA vs. AA), dominant (AG + AA vs. GG), over-dominant model (AA+GG vs. AG), were significantly associated with the decreased risk of cancer. Therefore, *MUC1* rs4072037 polymorphism was associated with decreased risk of gastric cancer in northern Iran.

The Prostate Stem Cell Antigen (*PSCA*) is expressed in the gastric epithelium of the isthmus area. This area includes immature epithelial cells. PSCA expression increases in prostate cancer but it is reduced in gastric cancer and gastric intestinal metaplasia [50, 51]. T allele carriers show lower transcriptional activity compared with the C allele carriers (5057C > T) in their gastric mucosa [31, 50]. T allele carriers have nine amino acids more than C allele carriers (natural protein of 114 amino acid residues) [52]. The association of *PSCA* (5057C > T) variant was described in Korea and Japan population for the first time and the T allele was reported as a risk factor for gastric cancer [31]. A study in Japan and Korea showed that 5057 T allele plays a more significant role in diffuse gastric cancer compared with the intestinal gastric cancer [31, 53]. Such results were also similar in the population of Poland and the United States [34]. Other studies in Caucasian and Chinese population also confirmed that this polymorphism is a risk factor for diffuse and intestinal gastric cancer [54].

In this study, *PSCA* rs2294008 polymorphism was not significantly associated with an increased risk of cancer in all genetic models (T vs. C (OR = 1.050, 95% CI: 0.652–1.693, *p* = 0.840); homozygous TT vs. CC (OR = 0.903, 95% CI: 0.327–2.492, *p* = 0.533); heterozygous CT vs. CC (OR = 1.228, 95% CI: 0.644–2.339, *p* = 0.533); dominant model CT + CC vs. TT (OR = 1.137, 95% CI: 0.420–3.080, *p* = 0.800); and recessive model CC vs. CT + TT (OR = 0.904, 95% CI: 0.505–1.620, *p* = 0.735) (Table 5). Therefore, *PSCA* rs2294008 polymorphism was not associated with increased risk of gastric cancer in Northern Iran.

## Conclusion

In conclusion, this study evaluated the effect of *MUC1* (rs4072037) and *PSCA* (rs2294008) polymorphisms on GC risk in the Northern Iran population. Data from this study revealed that the *MUC1* rs4072037 polymorphism was significantly associated with decreased risk of gastric cancer. However, the results could be important, especially for the interpretation of a genetic variant, which cause susceptibility to gastric cancer, and could be used as diagnostic markers in gastric cancer. In addition, *PSCA* polymorphism did not show any relation to gastric cancer in our findings probably because of the type of gastric cancer or the small sample size.

## Data Availability

The datasets used and/or analyzed during the current study are available from the author for correspondence upon reasonable request.

## Abbreviations

GWAS: Genome-Wide Association Studies
MUC1: Mucin 1
PSCA: Prostate Stem Cell Antigen
FFPE: Formalin fixed paraffin-embedded
TA-MUC1: tumor-associated MUC1
GPI: Glycosylphosphatidyl inositol
SNP: Single nucleotide polymorphism
PCR: *Polymerase chain reaction*
RFLP: Restriction fragment length polymorphism
*H. pylori*: Helicobacter pylori
